# Retroperitoneal Hemorrhage from Adrenocortical Carcinoma as a Poor Prognostic Factor

**Published:** 2008-03

**Authors:** Anna A. Kasperlik-Zaluska, Wojciech Zgliczynski, Rafal Z. Slapa, Andrzej Cichocki

**Affiliations:** 1 *Department of Endocrinology, Center for Postgraduate Medical Education, Warsaw, Poland;*; 2 *Department of Imaging, Medical University of Warsaw, Warsaw, Poland;*; 3 *Department of Surgery, Maria Sklodowska-Curie Cancer Center and Institute of Oncology, Warsaw, Poland*

**Keywords:** adrenal cancer, retroperitoneal hemorrhage, steroids

## Abstract

In most patients, adrenocortical carcinoma is diagnosed at an advanced stage of the disease. A sudden retroperitoneal hemorrhage may sometimes be the first symptom of the adrenal cancer. We describe four patients with adrenocortical carcinoma diagnosed during a retroperitoneal hemorrhage. A clinical analysis suggests that such a complication is a poor prognostic factor.

## INTRODUCTION

The detectability of adrenal cancer has significantly improved in recent years due to the development of new imaging techniques. Unfortunately, most of these tumors are identified at the locally invasive or metastatic stage (1). Rare cases manifest themselves by a sudden lumbar pain, followed by shock caused by retroperitoneal hemorrhage from a ruptured adrenal cancer. In the medical records of the Department of Endocrinology (Center for Postgraduate Medical Education, Warsaw), of 180 patients with adrenal carcinoma, there were four cases where such an incident was the first symptom of the disease.

## CASE REPORTS

### Case 1

A 12-year-old girl, after physical exercise at school, had a sudden pain in the right lumbar area accompanied by a brief lack of consciousness and tachycardia, followed by progressive weakness. Emergency surgery was indicated by a gradual fall in the hematocrit. A tumor of the right adrenal, 10 cm in diameter, and a retroperitoneal hemorrhage from the right adrenal vein were the main intraoperative findings. The histopathological diagnosis was adrenocortical carcinoma. After the operation the patient felt well. However, four months later signs of Cushing’s syndrome appeared (moon face, overweight, acne, purple striae, hirsutism, and hypertension). At admission to the Department of Endocrinology the girl was 160 cm in height and weighed 66 kg. Computed tomography (CT) revealed an abnormal mass (6 cm in size) at the upper pole of the right kidney; multiple metastatic tumors were identified in the lungs by a routine x-ray examination. An increase in the daily urinary excretion of steroids was also noted: 17-hydroxycorticosteroids (17-OHCS) - 30.4 mg (normal, 2.2-7.0), 17-ketosteroids (17-KS) - 44.4 mg (normal, 3.5-15.0). During a second operation (7 months after the first) the recurrent adrenal tumor and a metastatic mass in the omentum were removed. Therapy with aminoglutethimide (an adrenal inhibitor) was introduced - 1.0 g daily for three months - together with mitotane (LYSODREN - Bristol) in a long-term program. The daily doses of mitotane ranged from 3.0 to 8.0 g. Clinical remission, associated with a return of steroid excretion to normal values (17-OHCS - 3.0 mg/24 h, 17-KS - 2.4 mg/24 h) and a reduction in the size and number of pulmonary metastatic tumors, was reached within about five months. After two years, androgenization reappeared and pulmonary as well as abdominal carcinomatous recurrences progressed. The patient died about 2.5 years after the first surgical intervention. This patient has been registered in the IPACTR (2).

### Case 2

A 49-year-old woman was admitted to the Department of Endocrinology due to a recurrent adrenocortical carcinoma with invasion of the vena cava inferior and metastatic tumors in the liver. Three years previously she had been submitted to emergency surgery because of severe right abdominal pain followed by anemia and shock. An enormous retroperitoneal hematoma, penetrating to the peritoneal cavity (peritoneal rupture) was the initial surgical finding. After evacuation of the blood, a right-sided adrenal tumor (6 cm in diameter) and the right kidney (due to a lesion of the renal vein) were removed. Histopathologically the tumor was identified as adrenocortical carcinoma with hemorrhagic and necrotic areas. Shortly before the surgery, diabetes mellitus and hypertension were diagnosed. Normal 17-OHCS and 17-KS values were recorded after the operation. Mitotane was administered in doses ranging from 3.0 to 8.0 g daily. After the first year of therapy, characterized by partial clinical remission, the abdominal mass progressed and left-sided pulmonary metastatic tumors appeared. Chemotherapy (Cisplatin) was introduced, simultaneously with the administration of Mitotane, but without success. The patient died after two years of follow-up.

### Case 3

**Figure 1:**
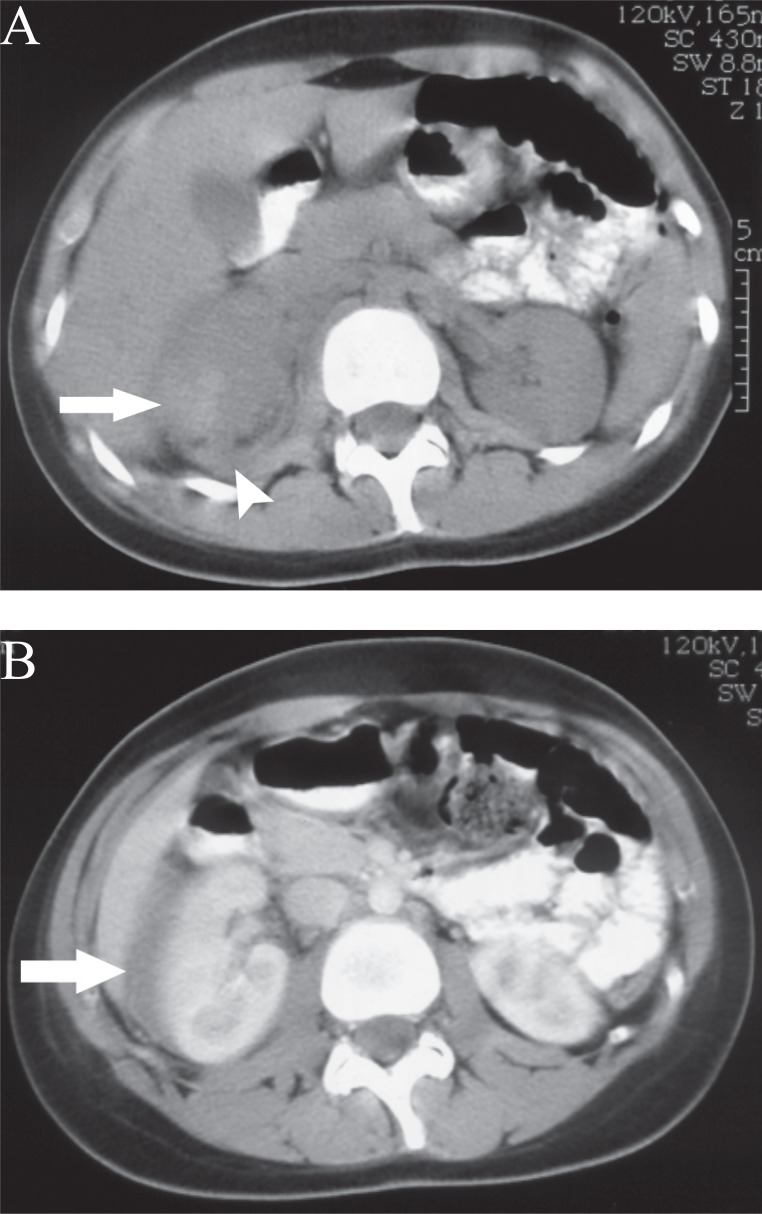
CT of right adrenal carcinoma. **(A)** On non-enhanced CT the hyperdense focus in the tumor (arrow) is seen. Extraadrenal haemorrhage (arrow head) is almost isodense with the tumor and with edema (arrow) surrounding the kidney seen on **(B)**, that is contrast-enhanced CT.

**Figure 2:**
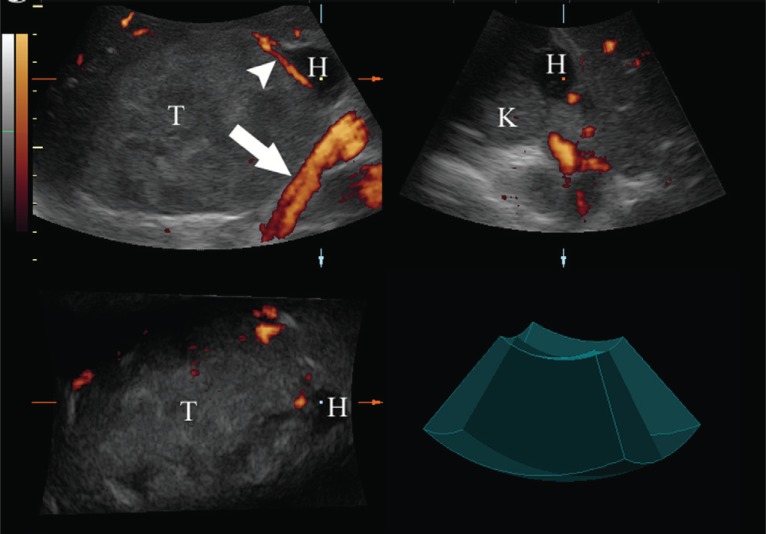
Three-dimensional sonography with power Doppler of the right adrenal carcinoma (T). Multiplanar reformation mode (MPR) showing the structures in 3 orthogonal planes. Two arteries (arrow) to right kidney and dominant inferior adrenal artery (arrow head) are visible. The extraadrenal haemorrage (H) is hypoechoic and is adhering to the kidney (K).

**Figure 3:**
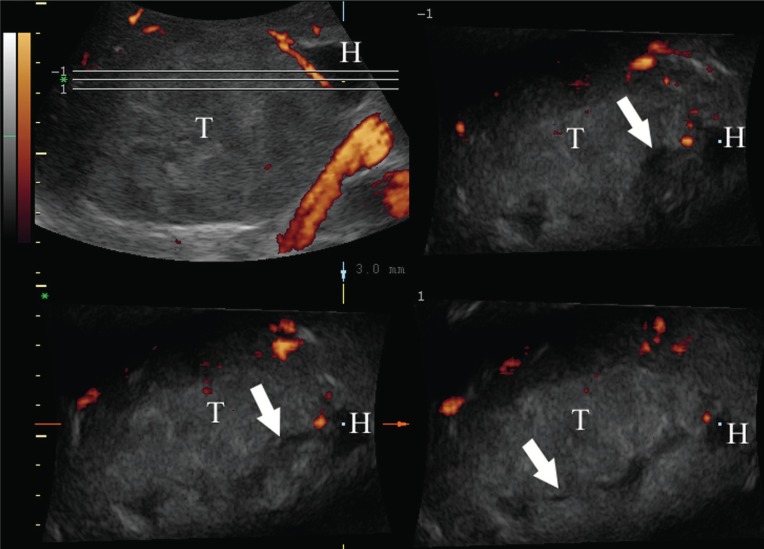
Three-dimensional sonography with power Doppler of the right adrenal carcinoma (T). Tomographic ultrasound imaging – TUI – is a postprocessing option enabling presentation of volume data on consecutive slices like with CT. On coronal pilot image (upper left) the position of consecutive slices in plane parallel to the ultrasound probe is marked. The extraadrenal haemorrage (H) is continuous with hipoechoic intratumoral haemorrage (arrows) seen in plane parallel to the ultrasound probe, which is unique for 3D sonography.

A young woman, aged 21 years, was admitted to our department because of a right adrenal tumor, found incidentally two weeks before, during routine ultrasonography after an emergency appendectomy for acute appendicitis. CT revealed a non-homogenous tumor of the right adrenal gland, 7 cm in size, and enlarged paracaval lymph nodes. No features of adrenal hyperfunction were observed. In hormonal examinations, slightly increased serum cortisol levels of 28 μg/dL (normal range: 5-25), and androstendione elevated to 475 ng/dL (normal: 85-270) were recorded. On the third day of observation the patient experienced sudden pain in the right lumbar area. A hemorrhage into the adrenal tumor was suspected and this was confirmed by three-dimensional sonography and CT (Figs. 1, 2 and 3). Surgery revealed a retroperitoneal hemorrhage and adrenal tumor with a hemorrhagic, gelatinous focus. The size of the subsequently removed tumor was 10 × 9 × 6 cm. On histopathological examination, adrenocortical carcinoma was diagnosed, with hemorrhagic and necrotic areas constituting 50% of the tumor. Invasion of vessels and a ruptured capsule of the tumor were found. The patient was treated with mitotane (4.0 g daily) and chemotherapy (cisplatin, vepezid). However, a large recurrent tumor was identified in the retroperitoneum following the first cycle of this therapy. She died five months after the tumorectomy. This patient has been described previously (3).

### Case 4

A 65-year-old woman was observed as an outpatient, admitted to our clinic (Department of Endocrinology) four weeks after emergency surgery for a retroperitoneal hemorrhage, with evacuation of 3.5 L of blood and removal of a tumor of the left adrenal gland. Her past history was a sudden lumbar pain in the left side with progressive weakness followed within six hours by shock. CT revealed a retroperitoneal, non homogenous tumor. Histopathology identified an 11 × 8 × 4.5 cm sized adrenocortical carcinoma with large necrosis of the tumor. Hormonal measurements performed after the surgery were within normal limits. Mitotane was administered, 4.5 g daily. Over the next six months, metastatic tumors were found in the left lung, as well as in the second lumbar vertebral body, with progressive paresis of the legs. The patient died two weeks after an orthopedic operation.

## DISCUSSION

Adrenocortical carcinoma is a rare tumor, representing approximately 0.02% of all cancers (4, 5). Most patients are diagnosed in a locally advanced or metastatic stage of the disease (6), i.e., MacFarlane’s classification stage III or IV (7), mainly following incidental findings during imaging examinations. In the Department of Endocrinology registry there have been 180 patients with adrenal cancer and 115 of them (64%) were diagnosed as incidentally-found adrenal tumors (adrenal incidentalomas). Thus, less than half were diagnosed due to endocrinological manifestations. A retroperitoneal hemorrhage revealing the existence of an adrenal cancer is a life-threatening event. It is a rare complication of adrenal cancer and was not described in two recent reviews concerning the diagnosis and treatment of adrenocortical carcinoma (8, 9). In our observations, a sudden lumbar or abdominal pain and a progressive decrease in the erythrocyte number, followed by shock, were the most characteristic features of this potentially fatal event. Emergency surgery is a life-saving procedure in such cases.

In none of our four patients were typical somatic signs of hypercorisolism or hyperandrogenism apparent prior to the hemorrhagic incident. The development of Cushing’s syndrome during the phase of carcinomatous dissemination in the patient No. 1 was a rare observation. Diabetes mellitus and hypertension preceding the hemorrhage in the patient No. 2 were probably the first symptoms of metabolic and vascular disorders due to adrenal carcinoma.

In all four patients the adrenal cancer followed an aggressive course, with rapid development of local recurrences and distant metastases. In case No. 3 a fulminant course of the adrenal cancer caused death within about six months following the hemorrhage, despite immediate mitotane administration and chemotherapy. Massive dissemination of neoplastic cells during such a severe vascular incident was probably responsible for the extensive local invasion and metastatic process in all of these patients.

In our experience (10) and in the opinion of others (11), stable remission may be obtained in some patients with tumors at stages III or IV of MacFarlane’s classification following mitotane administration and chemotherapy (10, 11, 12, 13). Long-term observations of the remaining group with adrenal cancer revealed that early administration of mitotane, immediately after surgery, resulted in a better prognosis than the same treatment introduced with a delay of more than two months following surgery. An analysis of 59 patients treated with mitotane - out of 82 patients observed up to the year 2000 - indicated that there was 56% survival in the group of 32 patients given early treatment with mitotane vs 22% in the group of 27 patients, in which mitotane administration was delayed (12). However, mitotane did not improve the prognosis in patients with a retroperitoneal hemorrhage in their past history.

In conclusion, a retroperitoneal hemorrhage due to an adrenocortical cancer should be considered as a bad prognostic factor.
